# Physical quality of gluten‐free doughs and fresh pasta made of amaranth

**DOI:** 10.1002/fsn3.3301

**Published:** 2023-03-14

**Authors:** Tanja Lux (née Bantleon), Frauke Spillmann, Frederike Reimold, Adam Erdös, Annekathrin Lochny, Eckhard Flöter

**Affiliations:** ^1^ Technische Universität Berlin, Institute for Food Technology and Food Chemistry Department of Food Processing Technology Berlin Germany; ^2^ Institute for Agricultural and Urban Ecological Projects (IASP) affiliated to Humboldt Universität Berlin Berlin Germany; ^3^ University of Applied Sciences Bremerhaven, Food Technology of Animal Products Bremerhaven Germany

**Keywords:** Amaranth, dough properties, gluten‐free pasta, sodium alginate, texture properties

## Abstract

Pasta is one of the most consumed foods in the world. Therefore, the development and investigation of the quality parameters of fresh gluten‐free pasta made from amaranth was the subject of this study. For this purpose, different doughs (amaranth flour: water 1:2, 1:4, 1:6, 1:8, 1:10) were heat‐treated and sodium alginate (1.0 and 1.5%) was added. The pasta was produced by extrusion into a 0.1 M calcium L‐lactate pentahydrate‐containing bath. Both the dough and the pasta were examined. The doughs for its viscosity properties, water content, and color and the pasta for its firmness, color, water content, water absorption, cooking loss, and swelling index. The pasta was cooked for 5, 10, and 15 min for the cooking quality study. A higher alginate content of 1.5% and a higher proportion of amaranth flour resulted in a significant difference in color, water content, and shear‐dependent viscosity of the dough (*p* < .001). It was also found that both doughs with amaranth flour‐water content of 1:2 and 1:10 had significant effects on processing properties and pasta quality, especially on firmness, swelling index, and cooking loss. For the doughs with a 1:2 ratio, the high flour content resulted in very soft pasta, and for the doughs with a 1:10 ratio, the high‐water content resulted in very firm pasta with a smooth, watery surface. Overall, cooking loss, swelling index, and water absorption were low for the pasta with 1.5% alginate. Even with cooking times of 15 min, the pasta retained its shape.

## Graphical abstract

The range of gluten‐free foods, such as gluten‐free pasta, has grown steadily in recent years. However, fresh pasta is not widely available in this area. Therefore, in this research work, a gluten‐free fresh pasta was successfully developed from the nutritionally valuable pseudocereal amaranth by successfully replacing gluten with the hydrocolloid sodium alginate.

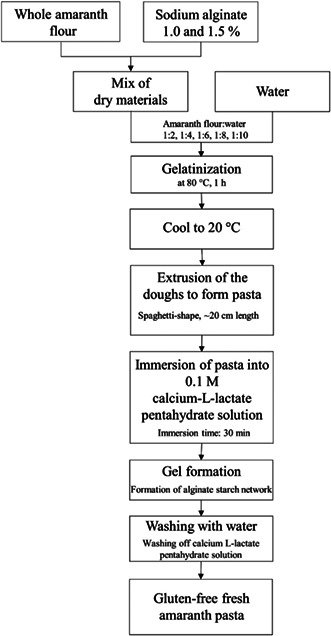

## 1 INTRODUCTION

Pasta is one of the most consumed foods in Europe, due to its easy preparation, handling, and storage conditions (Marconi & Messia, 2012). Pasta made from durum wheat semolina is also popular because of its sensory and technological properties (Kill & Turnbell, 2001). Due to the increasing number of consumers affected by gluten or other wheat‐based food intolerances, the demand for the substitution of wheat in pasta with gluten‐free raw materials is growing (Schoenlechner, 2017). Those affected by such intolerance must follow a lifelong gluten‐free diet.

The pseudocereal amaranth is naturally gluten‐free, highly nutritious e. g. high content of fiber, starch, and protein compared to cereals such as wheat and, therefore, predestined for use in gluten‐free foods. Due to its nutrient composition, amaranth has potential health benefits (Belton & Taylor, 2002). The production of pasta from and with amaranth has been studied in some works (Cárdenas‐Hernández et al., 2016; Fiorda et al., 2013; Schoenlechner, 2017). In these research works, mainly dried pasta was produced, some of which also contained gluten for structural stabilization. However, corn, rice, or potatoes are often used to make gluten‐free dried pasta. Pseudocereals have so far only been partially used as an ingredient for gluten‐free pasta (De Arcangelis et al., 2020; Marconi & Messia, 2012; Motta Romero et al., 2017). To date, there are a few known research papers that address the issue of gluten‐free fresh pasta. These often consist of millet, rice, soya, or buckwheat (Cordelino et al., 2019; Martínez et al., 2017; Sanguinetti et al., 2015; Tyl et al., 2020).

It is an enormous technological challenge to replace gluten, as it is an important structure‐forming protein in bakery and pasta products. By forming a strong protein network, the starch is retained in the pasta during cooking. When gluten‐free raw materials are used, the viscoelastic properties of the dough, cooking quality, and sensory characteristics may also change (De Arcangelis et al., 2020). Therefore, substances imitating these properties are needed. Nowadays food products made of gluten‐free grains are often mixed with starches and hydrocolloids to imitate the structure of gluten (Bastos et al., 2016; Coronel et al., 2021; Turkut et al., 2016). An example of hydrocolloids used in this context are alginates. They are often used in the food industry for its stabilizing, solidifying, and gel‐forming properties. Alginate is the salt of alginic acid found in the cell walls of the brown algae Phaeophycea (Roberts et al., 2000). Sodium alginate can form a high‐temperature‐resistant gel when combined with divalent cations. Calcium ions are most frequently used for this purpose. The gel strength depends, among other factors, on the proportion of polyguluronate in the alginate. During gel formation, two polyguluronate chains, each folded, bind calcium cations between the two chains. This binding is known as the so‐called “egg box model.” The polymannuronate, on the other hand, forms polyelectrolyte bonds with the calcium ions, which are soluble in water. Gel formation occurs immediately and irreversibly after the addition of calcium cations to alginate (Roberts et al., 2000; Skurtys et al., 2014).

Sodium alginate was used in the present work to stabilize the amaranth flour and form the pasta. In other studies, the effects of alginates in binary systems with different flours and starches have also been investigated (Chrastil, 1991; Jang et al., 2015; Liu et al., 2020; Motta Romero et al., 2017; Roberts et al., 2000). This work aimed to produce pregelatinized doughs with different proportions of amaranth flour and water. Two different concentrations of sodium alginate were added to the different proportions of amaranth flour. The pasta was formed with a calcium‐induced cross‐linking bath. In this study, both the properties of the different doughs and the cooking properties of the different kinds of pasta were investigated. The doughs were analyzed for its shear‐dependent viscosity, water content, and color. For the pasta, the firmness, color, water content, water absorption (WA), volume increase (SI), and cooking loss (CL) of the uncooked and cooked pastas were measured.

## 2 MATERIALS AND METHODS

### 2.1 Materials

Amaranth grains from *Amaranthus caudatus ‐ Oscar blanco* from De Guste Group Sac (Cotahuasi, Peru) were used to produce suspensions and pastas. The amaranth grains were stored in a cool, dry, and dark place. Hierl Naturkost GmbH (Stallwang, Germany) carried out the grinding of the grains to a fine flour, with a Zentrofan mill model C 25/C 75 (Zentrofan‐Mühlenbau UG. Überlingen am Bodensee, Germany). The flour had a water content of 12.51% ± 0.73, determined by infrared moisture meter IR 35 (Denver Instrument GmbH, Göttingen, Germany), and a water activity of 0.56 ± 0.02. The amaranth flour had an amylose content of 2.57% ± 0.03 and was determined using the Amylose/Amylopectin Assay Kit (Megazyme, Megazyme International, Ireland). Sodium alginate NA4012 (C. E. Roeper GmbH, Hamburg, Germany) was used for structural stabilization. For gel formation, calcium L‐lactate pentahydrate from Carl Roth was used (Carl Roth GmbH & Co. KG).

### 2.2 Dough and pasta preparation

**FIGURE 1:**
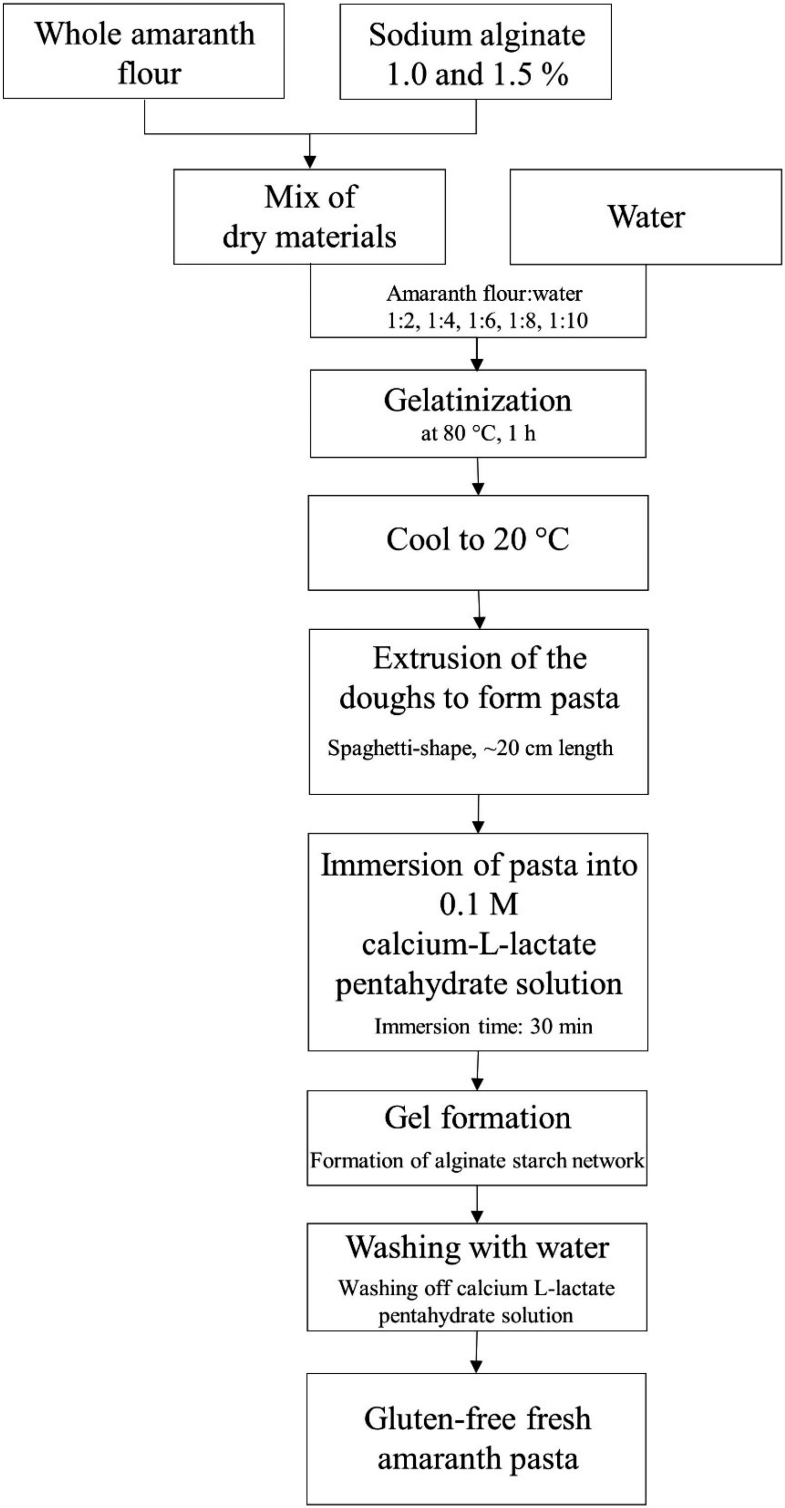
Flow chart of gluten‐free fresh amaranth pasta production.

The pasta‐making process is shown in a flow chart in Figure 1. The dry materials were premixed. In the process, 1.0 or 1.5 wt% alginate was added to the whole amaranth flour. This mixture was then combined with water in the following proportions: 1:2, 1:4, 1:6, 1:8, 1:10 amaranth flour: water. The suspensions were heated in a Thermomix TM5 (Vorwerk Elektrowerke GmbH & Co. KG) for 60 min at 80°C and 300 rpm (Lux et al., 2021).

For pasta preparation, the suspensions were extruded into a 0.1 M calcium L‐lactate pentahydrate (Carl Roth GmbH & Co. KG, Karlsruhe, Germany), which had a temperature of approximately 20°C. Extrusion was conducted by a manual press with a perforated sieve insert and formed spaghetti‐like pasta (Model 61102260; Westmark GmbH). For gel formation, the pasta was left in the 0.1 M calcium L‐lactate pentahydrate lactate bath for 30 min, then washed with deionized water and dried on the surface. To analyze the pasta quality, the samples were afterward cooked in deionized water at 100°C for 5, 10, and 15 min, respectively. These long cooking times were chosen to examine whether quality losses occur after longer cooking times. The doughs as well as the pasta in an uncooked and cooked state were examined. Each dough and pasta mixture was produced and tested at least three times (*n* = 3).

### 2.3 Shear rate measurements of doughs

A controlled shear rate test of the individual doughs was investigated with a rheometer Physica MCR 302 (Anton Paar GmbH) with the concentric cylinder CC27/ P1 (DIN standard, *d* = 0 mm). The shear rate was increased from 0.1 to 100 1/s, continuously at 20°C. Each measuring point was held for 2 s and recorded with the software RheoPlus V3.62. The results provide information about the handling and processing of the suspensions, such as the force to be applied during extrusion through the manual press. The Herschel‐Bulkley model was applied for fitting all experimental curves. This model describes the data of pseudoplastic (*n* < 1), dilatant (*n* > 1), or Newtonian fluids (*n* = 1) by considering possible yield stress. The regression model is described with the following formula (Rao, 2007):

$$\text{Herschel}-\text{Bulkley}:\tau =\tau_{0}+k^{*}{\dot{\gamma }}^{n}$$

τ for shear stress (Pa), τ_0_ for yield stress (Pa), *k* for consistency coefficient (Pa s^*n*^), $\dot{\gamma }$ for shear rate (s^−1^), and *n* for flow index (dimensionless).

All samples were investigated in triplicates (*n* = 3).

### 2.4 The water content and color determination of amaranth doughs and uncooked pasta

To determine a change in water content of amaranth doughs and pasta with different flour and alginate ratios, a volumetric water determination was carried out by volumetric Karl‐Fischer titration (KFT). This method is based on chemical water determination. The titrator apparatus 870 KF Titrator plus (Deutsche Metrohm GmbH & Co. KG) is equipped with a 10 mL dosing system. As a reagent, Hydranal‐Methanol Rapid and Hydranal‐Composite 5 (Sigma‐Aldrich Chemie GmbH) as titration solutions were used. For the determination of titer, Karl‐Fischer‐Rotihydroquant standard sodium tartrate dihydrate (Carl Roth GmbH & Co. KG) was applied. Due to the high‐water content of the samples, 0.0200 g ± 0.0001 were weighed. Sample comminution was performed by T25 digital Ultra‐Turrax (IKA‐Werke GmbH & Co. KG). Samples were performed in triplicates (*n* = 3).

The color change of the doughs, the uncooked and cooked amaranth pasta, were measured with CIELAB parameters (L*a*b*) at D65 illumination conditions with a Chroma Meter CR 200 (Konica Minolta Sensing Europe B.V.). For each measurement 20.0 g ± 1.0 of the samples was placed in a clear, flat bottom cylinder with 20 mm in diameter. The measurement was performed *n* = 5. From the measured data, the yellowing index (YI) was calculated with the following equation (Rufián‐Henares et al., 2006):

$$\mathrm{YI}=142.86\;b^{*}/L^{*}$$

L*‐value for brightness, and b*‐value for blue/ yellow opponents.

### 2.5 Characterization of texture properties of pasta

The texture of amaranth pasta was analyzed using a Textural Analyzer BT1 FR2.5TN.140 with a 2.5 kN load cell (Zwick‐Roell GmbH & Co. KG). Samples were rested for 15 min after cooking before being measured. Approximately 100 mL of each pasta sample was added to an Ottawa Texture Measuring System (OTMS cell), imitating chewing. This measurement tests the strength of the sample by compression and extrusion. A punch was pressed into the sample at a speed of 60 mm/min. An extrusion plate with 2.5 mm wide cut edges was located under the sample. The measurement was stopped after a sample deformation of 80%, and the results are expressed as maximal extrusion force in *N*. Data are mean values of three measurements (*n* = 3) from three different cooking replications.

### 2.6 Cooking quality of the pasta: Water absorption, cooking loss, and swelling index

The water absorption of 10 g amaranth pasta during cooking was expressed by water absorption (WA, g/100 g) as:

$$\mathrm{WA}=\frac{\text{weight of cooked noodles}-\text{weight of}\;\mathrm{raw}\;\text{noodles}}{\text{weight of}\;\mathrm{raw}\;\text{noodles}}∙100$$

Cooking loss (CL, g/100 g) was determined according to Approved Method 66–50 (AACC, 2000). Residues of cooking water were determined gravimetrically. 25 mL of cooking water was dried at 105°C to constant mass and analyzed.

$$\mathrm{CL}=\frac{\text{weight of dried residue in cooking water}}{\text{weight of}\;\mathrm{raw}\;\text{noodles}}$$

The increase in the volume of the pasta is expressed by the swelling index (SI, g_water_/g_dry pasta_). 10 g of amaranth pasta was weighed after cooking and dried at 105°C to a constant weight. The swelling index was determined as:

$$\mathrm{SI}=\frac{\text{weight of cooked noodles}-\text{weight of cooked noodles after drying}}{\text{weight of cooked noodles after drying}}$$

WA, CL, and SI were determined in triplicates for the suspensions (*n* = 3).

### 2.7 Statistical analysis

The statistical analysis was carried out with SigmaPlot (Sigmaplot 11.0 for windows; Systat Software Inc.). Significant differences between the samples of viscosity, water absorption, swelling index, and cooking loss were calculated using analysis of variance (ANOVA). Mean values were compared by Tukey's test with *p* < .05. Results are shown with the mean value including the standard error of means. The Pearson correlation coefficient was used to calculate the linear relationship between the two variables. The rheological data were analyzed with the software RheoPlus V3.62 (Anton Paar GmbH). Data are mean values of at least three measurements from three different batches of pasta produced.

## 3 RESULTS AND DISCUSSION

### 3.1 Amaranth dough properties

In the preparation of the doughs, the starch contained in the amaranth flour was pregelatinized, thus improving the rheological properties of doughs and the cooking quality of pasta (Marconi & Messia, 2012). Amaranth starch gelatinizes between 62 and 68°C, depending on the available water content (Arendt & Bello, 2008; Gamel et al., 2005). The temperature of 80°C applied to the flour blends in this study guarantees gelatinization and partial denaturation of some amaranth proteins, which also contributes to the strength of the pasta (Janssen et al., 2017; Lux et al., 2021). In the starch gelatinization process, the starch granules absorb water through hydrothermal treatment until the swollen granules break and a gel with greatly increased viscosity is formed. During the cooling process of gelatinization, retrograded starch is formed. This is thermally stable, represents a form of undigested starch and, in turn, has beneficial nutritional effects similar to those of dietary fiber (De Arcangelis et al., 2020). Amaranthus caudatus starch consists largely of amylopectin and only between 4.7% and 12.5% of amylose (Singh et al., 2014; Velásquez‐Barreto et al., 2021). Due to the low amylose content, amaranth starch partly behaves differently in its properties. For example, the retrogradation of the starch takes place more slowly due to the higher amylopectin content. This is also the reason why amaranth seems to be less suitable for the production of pasta, for example (Shevkani et al., 2021). The presence of other polymeric substances, such as proteins, fibers, or hydrocolloids, also affects the properties and quality of the final product by interacting with the starch. This can be seen by the interaction of alginate and starch in the present study. From the different doughs made from different parts of water and amaranth flour added with sodium alginate, the shear‐dependent behavior, the water content (WC), and yellowness index (YI) were analyzed. Visually, clear differences in the doughs were identified. In terms of color, the doughs with an increased amaranth flour content appeared darker. This was confirmed by the YI in Table 1. With decreasing amaranth flour content, the YI also decreased, and the doughs appeared brighter. The doughs with 1.5% alginate showed lower YI compared to 1.0% (*p* < .001) and also showed that the doughs had different viscosities.

**TABLE 1:** Dough properties of different water to amaranth flour ratios (1:2, 1:4, 1:6, 1:8, 1:10) with 1.0% and 1.5% alginate added.

Sample code	η_10 1/s_ [Pa s]	η_100 1/s_ [Pa s]	τ_0_ [Pa]	k [Pa s^n^]	*n*	*R* ^2^	Water content [g/100 g]	YI
1:2, 1.0%	118.71 ± 3.06^abc^	18.31 ± 3.30^abc^	406.12	295.51	0.39	0.79	70.16 ± 0.63^abc^	32.64 ± 0.93^ab^
1:4, 1.0%	32.39 ± 2.58^ac^	6.26 ± 0.25^abc^	109.29	68.46	0.47	0.92	82.50 ± 0.21^a^	23.48 ± 0.43^ab^
1:6, 1.0%	17.19 ± 0.50^b^	4.10 ± 0.05^a^	58.95	29.10	0.55	0.99	80.51 ± 0.11^b^	20.01 ± 0.76^ab^
1:8, 1.0%	11.06 ± 0.19^a^	2.71 ± 0.10^b^	42.65	17.33	0.57	0.99	83.37 ± 0.78^c^	15.07 ± 0.42^a^
1:10, 1.0%	6.69 ± 0.12^c^	1.84 ± 0.14^c^	22.31	10.32	0.61	0.99	89.38 ± 1.19^abc^	11.44 ± 0.71^b^
1:2, 1.5%	155.49 ± 10.06^klmn^	15.83 ± 1.28^klm^	693.72	320.51	0.31	0.58	66.04 ± 0.21^klmn^	31.19 ± 1.09^k^
1:4, 1.5%	51.43 ± 1.93^klmn^	10.29 ± 2.02^k^	173.53	96.60	0.50	0.91	79.91 ± 0.03^kmn^	24.58 ± 0.46^k^
1:6, 1.5%	25.89 ± 1.41^l^	5.05 ± 0.07^l^	85.93	45.532	0.51	0.94	86.59 ± 0.15^lmn^	19.38 ± 0.79^k^
1:8, 1.5%	16.39 ± 1.24^m^	3.88 ± 0.07^m^	59.72	28.53	0.55	0.97	81.07 ± 0.84^L^	15.84 ± 0.40^k^
1:10, 1.5%	11.98 ± 0.21^n^	2.91 ± 0.17^klm^	41.42	18.64	0.59	0.98	89.72 ± 0.26^n^	12.18 ± 0.66^k^

*Note*: Data are mean values ± standard deviation. Same letters in the same columns indicate significant differences (*p* < .05). Significant differences were compared within dough properties with the same alginate concentrations.

The dough properties are decisive for its processing success: they must be neither too solid nor too liquid. All samples showed shear‐thinning fluid behavior according to the Herschel‐Bulkley model with *n* < 1. Both viscosity and water content (Table 1) were significantly affected by the alginate content (*p* < .001). The increase in viscosity was mainly due to starch gelatinization of amaranth flour and was observed with an increasing amaranth flour content of the doughs at the same alginate concentration. This was evident by an increase in the consistency coefficient with increasing flour content from 10.3 to 295.5 Pas^n^ for 1.0% and 18.6 to 320.5 Pas^n^ for 1.5% alginate, respectively (Table 1). As De Arcangelis et al. (2020) found, the small particle size of buckwheat starch granules has a high‐water absorption capacity. Due to the small size of amaranth starch granules (1–3 μm; Arendt & Bello, 2008), this effect is given here, too. As Liu et al. (2020) found in their study, a k‐values above 200 Pas^n^ did not exhibit suitable extrusion properties and could only be extruded with great force. The k‐values greater than 200 Pas^n^ occurred in the 1:2 doughs in this study with 295.51 Pas^n^ for 1.0% and 320.51 Pas^n^ for 1.5%, respectively (Table 1). The extrusion force to produce these pastas can be described as high in this study, which is in accordance with Liu et al. (2020), who studied the influence of alginate and rice paste. However, there must be sufficient water to completely gelatinize the starch contained in the system (Kong et al., 2009). It can be assumed that in the doughs with a high amaranth flour content as in the 1:2 doughs, the starch was not completely gelatinized due to the lack of water in the system, i. e. there was only partial gelatinization. This could also be confirmed by running a gelatinization profile using a rheometer (Data not shown). However, by heating the suspensions to 80°C, the alginate concentration also contributes to the viscosity increase. The carboxyl and hydroxyl groups in the alginate structures enable a high‐water absorption, resulting in an increased swelling capacity of starch granules (Jang et al., 2015; Rojas et al., 1999). All dough samples showed an increase in viscosity with an added alginate concentration of 1.5%. This may be due to the property of alginate to form hydrogen bonds with water molecules (Liu et al., 2020). The decreased *n*‐ and increased τ_0_‐value indicated that the amaranth doughs with the alginate content of 1.5% had stronger mechanical strength and better shape stability, as Mancini et al. (1996) and Peressini et al. (2018) also showed. They found that an increased amount of alginate binds more water, making the doughs firmer. With an increased shear rate, the viscosity of the binary starch‐alginate system decreased, as Roberts et al. (2000) found. The shear‐thinning behavior is characteristic of diluted starch concentrations. Increasing shear influenced most of the suspensions with a higher flour content (1:2 > 1:4 > 1:6 > 1:8 > 1:10), whereas the higher alginate content in the samples leads to higher total viscosity. There was a significant difference in measured viscosity values at 10 1/s for 1:2 (*p* < .001), 1:4 (*p* < .001), and 1:6 (*p* = .028) and for 100 1/s of 1:2 (*p* = .003) as well as 1:4 (*p* = .01) doughs within the alginate concentrations 1.0 and 1.5%. Extremes were found in the 1:10 and 1:2 doughs. In the 1:10 doughs, water was present in excess, and the starch content of amaranth flour was lowest in comparison. These doughs were thin and liquid, resulted in a low viscosity and a high‐water content (Table 1). In contrast, a lack of water was observed in the 1:2 doughs. The dough was firm, and it can be assumed that due to the lack of water, the starch was not completely gelatinized, as well as the alginate only formed a loose bond. The results of the water contents are shown in Table 1. Generally, the water content increased with decreasing amaranth flour content. Significant lower water content was found in the samples with 1.5% alginate (*p* < .001). In general, the water content increased with decreasing amaranth flour content. A significantly lower water content was found in the samples with 1.5% alginate (*p* < .001).

### 3.2 Fresh amaranth pasta properties

#### 3.2.1 Texture analysis and water content of cooked amaranth pasta

**FIGURE 2:**
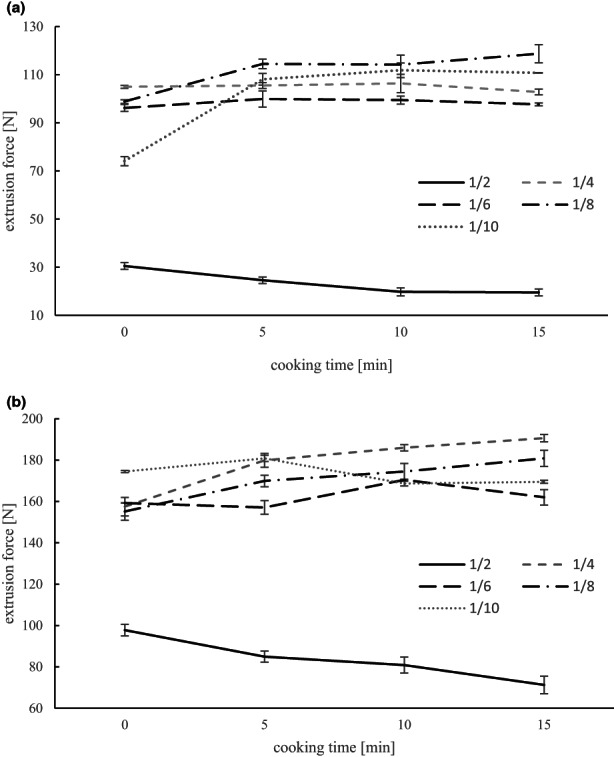
Firmness of fresh amaranth pasta made from amaranth flour and water (ratios 1:2, 1:4, 1:6, 1:8, 1:10) with a) 1.0 or b) 1.5% alginate by extrusion into an 0.1 M calcium lactate bath; uncooked (0 min) and cooked (5, 10, and 15 min). Data are shown as extrusion force in N, measured with Ottawa Texture Measuring System (OTMS).

**FIGURE 3:**
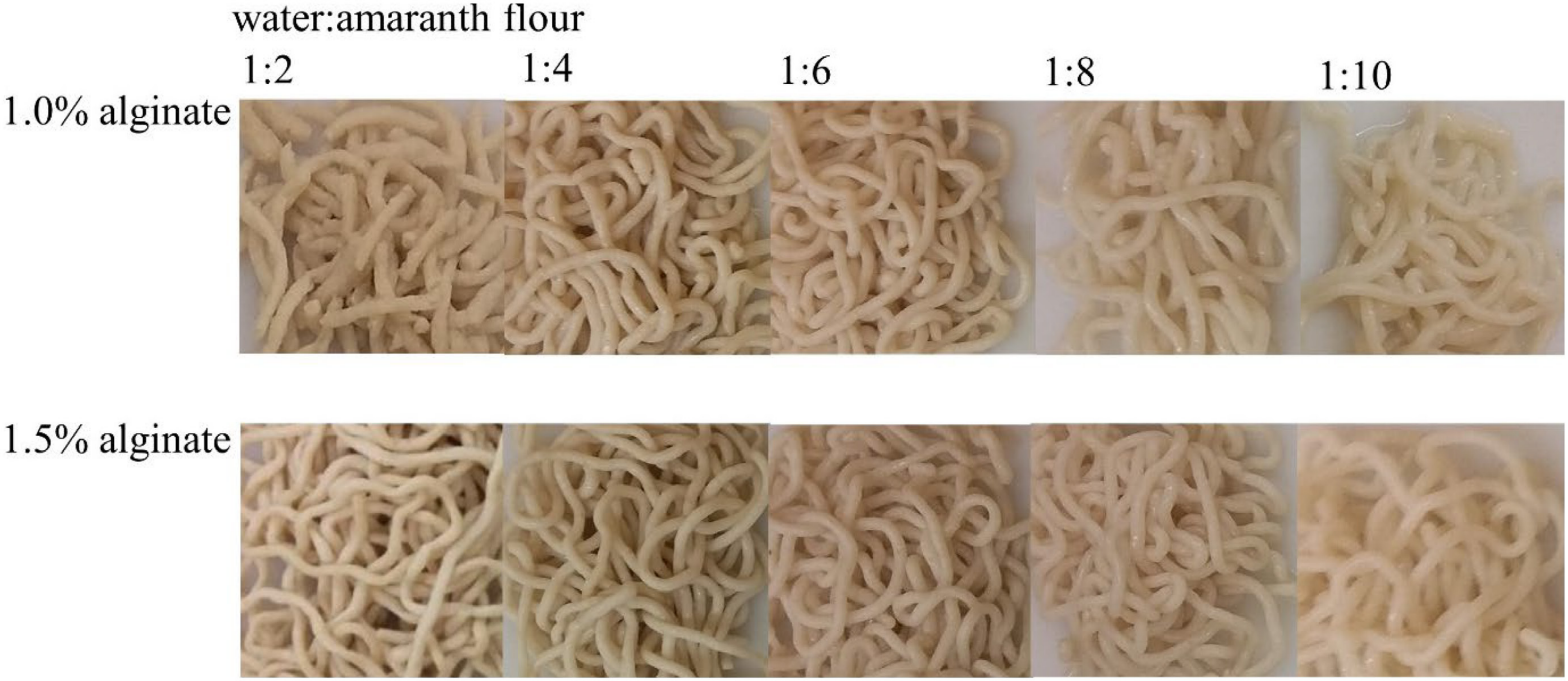
Uncooked fresh pasta made from amaranth flour and water (ratios 1:2, 1:4, 1:6, 1:8, 1:10) with 1.0% or 1.5% sodium alginate by extrusion into an 0.1 M calcium lactate water bath.

The firmness of pasta is an essential factor in the consumer's acceptance. The measured values show the simulation of the chewing behavior of the uncooked and cooked amaranth pasta by compression, measured with the OTMS cell (Figure 2a,b). To transfer the valuable ingredients of amaranth grains to the pasta, the highest possible flour content must be achieved. The 1:2 doughs had the highest flour and lowest moisture content in this study. However, the surface was rough and the texture soft (Figure 3), which negatively affected the quality of the pasta, especially its firmness properties. The high moisture content of the dough 1:10, on the other hand, resulted in pasta with a very tough consistency and a smooth, watery surface (Figure 3). The doughs with high‐water content (1:10) were very liquid but still resulted in firm pasta due to the alginate. Texture analysis of the pasta showed that with higher amaranth flour content, the firmness of the pasta decreased with decreasing amaranth flour content (Figure 2a,b). The exception was the 1:2 samples, which had the lowest firmness of all samples. There was a correlation between water content and firmness of uncooked pasta with 1.0% and 1.5% alginate (*r* = .75 and *r* = .82, respectively). Roberts et al. (2000) and Lubowa et al. (2020) found that the presence of starch and alginate in a system contributes to competition for the counterion calcium, thus increasing the calcium ion tolerance of the gels. Therefore, more calcium ions are required for the formation of firmer gels. As there is a significant difference between the alginate additions 1.0% and 1.5% for the firmness and the water content of the uncooked pasta (*p* < .001), this is in accordance with the study of Roberts et al. (2000). As in Chrastil (1991), it is not possible to precisely regulate the firmness of the gel by the amount of amaranth flour because there is no proportional dependence of the firmness.

A longer cooking time of the pasta partially led to an increase in firmness (Figure 2a,b). As Lubowa et al. (2020) found when making gluten‐free noodles with added alginate, adding alginate leads to improved cooking tolerance. However, it was noted that the firmness further decreased due to cooking in the 1:2 doughs. For these samples, it can be assumed that the starch is not completely gelatinized during the preparation of the suspensions, due to the insufficient amount of water. The ungelatinized starch begins to swell during the cooking of the pasta and partially destroys the gel structure, which leads to a softening in texture. As Liu et al. (2020) found, pasting viscosities decreased with increasing sodium alginate amounts. They attributed this to hydrogen bonds between starch molecules and sodium alginate. This cross‐linking limits the swelling of the starch grains during heating. When the starch grains are reheated by boiling, the hydrogen bonds are partly broken and the unswollen starch grains become gelatinized due to the presence of water. In contrast, the firmness of sample 1:4 1.5% alginate increased with increasing cooking time. Samples 1:8, 1.5%, and 1.0% as well as 1:10, 1.0% alginate samples had similar effects. It is assumed that in these samples the hydrogen bonds were increasingly loosened by heating, allowing water to escape from the pasta and thus making them firmer. For the 1:4, 1.0%, 1:6, 1.0 and 1.5%, and 1:10, 1.5% samples, minimal differences in firmness occurred due to boiling. An intact network appears to have been formed in these samples, so little change in firmness was observed. Also, a sensory ranking test of the pasta was performed (data not shown). There was also a sensory difference between the different flour and alginate contents of the pasta produced: Pasta with less alginate were perceived as softer, while noodles with little amaranth flour content were noted as firmer. Amaranth taste and odor were noted in all samples. Especially in samples 1:2 and 1:6, the texture was perceived as significantly different and as soft. These results thereby support the texture analysis measurements to a large extent.

#### 3.2.2 Color changes of amaranth pasta during cooking

The color of the pasta is an important factor for consumer acceptance. The L* (lightness) and b* (yellowness) values are important color attributes for pasta, which are directly related to its acceptability (Rayas‐ Durate et al., 1996). The color of the pasta is determined by the pigments in the raw materials. The YI of pasta should remain constant during cooking. However, the YI of amaranth pasta was significantly lower than that of wheat semolina pasta (YI for uncooked semolina pasta: 40.27 ± 1.05 (Teterycz et al., 2019)). The YI was calculated from the uncooked and cooked amaranth pasta to determine the change during cooking (Table 2a,b). There was a significant difference in YI between the uncooked pasta samples (*p* < .001), as the YI decreased with decreasing flour content. Pasta with higher amaranth flour content appeared visually yellowish‐brown, whereas the pasta with low flour content was whiteish (Figure 3). This was also reflected in a significant change in the a*‐value (red to green), which was in the positive, i. e. reddish, range for samples 1:2 and 1:4 at 1.0% and 1:2–1:6 at 1.5% alginate. As the amaranth flour content decreased, the a*‐value changed to a greenish range (data not shown). The color of the 1:2 pasta with 1.0% and 1.5% alginates was significantly darker compared to the other samples. The surface of the 1:2 pasta appeared porous. These samples were shorter, and the length decreased further with increasing cooking time. In addition, with increasing cooking time, the YI of all samples decreased significantly (*p* < .001). This was due to the increased cooking loss (CL; Table 2a,b) and further denaturation of the proteins (Janssen et al., 2017) contained in the amaranth flour. Samples with a lower proportion of amaranth flour (1:6, 1:8, and 1:10) showed less change in YI with longer cooking times, thus remaining more constant in its overall color shade. In addition, there was a significant difference between the samples with 1.0% and 1.5% alginate (*p* < .001). The YI of the uncooked pasta with 1.0% alginate was higher than those with 1.5%. This is in accordance with the study of Lee et al. (2008), who found that an increase in alginate content resulted in a decrease of L*‐ and an increase in b*‐value. Pasta with higher alginate content, therefore, appeared darker and greener. Overall, cooking affected the samples with 1.0% alginate more than the samples with 1.5% alginate. The results showed that the YI changes during cooking for all samples, but the intensity of the YI depends on the composition of the pasta.

**TABLE 2:** Quality parameters of fresh amaranth pastas (amaranth flour: water 1:2, 1:4, 1:6, 1:8, 1:10) with a) 1.0% and b) 1.5% alginate in uncooked and cooked state (5, 10, and 15 min).

(a) 1.0% alginate amaranth flour: water ratio	cooking time [min]	YI (*n* = 5)	WA [g/100 g] (*n* = 3)	CL [g/100 g] (*n* = 3)	SI [g/g] (*n* = 3)
1:2	Uncooked	30.07 ± 0.52	–	**–**	**–**
	5	29.84 ± 1.01	6.61 ± 2.12^ac^	1.79 ± 0.05^a^	3.44 ± 0.10^ac^
	10	27.12 ± 0.57	12.63 ± 1.71^cb^	1.97 ± 0.11^b^	3.79 ± 0.09^bc^
	15	26.87 ± 0.09	15.66 ± 1.73^a^	2.27 ± 0.06^a^	3.99 ± 0.08^a^
1:4	Uncooked	23.33 ± 0.12	–	–	–
	5	21.33 ± 0.46	7.66 ± 0.82^a^	1.44 ± 0.02^a^	5.24 ± 0.04^a^
	10	21.88 ± 0.58	9.03 ± 1.15^b^	1.77 ± 0.03^b^	5.55 ± 0.09^b^
	15	21.06 ± 0.48	9.94 ± 0.24^c^	1.48 ± 0.50^c^	5.79 ± 0.19^a^
1:6	Uncooked	21.30 ± 1.56	–	–	–
	5	19.01 ± 0.79	4.56 ± 1.75^a^	0.98 ± 0.14^ac^	8.14 ± 0.22^a^
	10	18.14 ± 0.79	6.16 ± 1.09^b^	1.43 ± 0.06^bc^	8.27 ± 0.18^b^
	15	17.01 ± 0.81	9.82 ± 2.42^c^	1.46 ± 0.20^a^	8.83 ± 0.41^a^
1:8	Uncooked	15.07 ± 0.62	–	–	–
	5	16.94 ± 0.62	8.56 ± 2.93^a^	1.49 ± 0.68^a^	9.12 ± 0.19^a^
	10	17.24 ± 0.74	9.30 ± 0.90^b^	1.26 ± 0.04^b^	9.47 ± 0.32^b^
	15	15.54 ± 0.57	12.07 ± 2.98^c^	2.09 ± 0.75^c^	9.70 ± 0.47^c^
1:10	Uncooked	13.98 ± 0.32	–	–	–
	5	16.66 ± 0.26	−10.92 ± 0.80^ab^	1.34 ± 0.08^a^	9.20 ± 0.25^a^
	10	17.29 ± 0.95	−14.95 ± 2.99^a^	1.30 ± 0.44^b^	9.11 ± 0.40^b^
	15	16.66 ± 0.26	−15.15 ± 0.94^b^	1.44 ± 0.15^c^	9.03 ± 0.08^c^

(b) 1.5% alginate amaranth flour: water ratio	Cooking time [min]	YI (*n* = 5)	WA [g/100 g] (*n* = 3)	CL [g/100 g] (*n* = 3)	SI [g/g] (*n* = 3)
1:2	Uncooked	27.84 ± 0.67	–	–	–
	5	28.85 ± 1.56	4.70 ± 2.50^a^	1.52 ± 0.77^a^	3.21 ± 0.03^ac^
	10	26.97 ± 1.31	7.98 ± 2.57^a^	1.73 ± 0.19^b^	3.47 ± 0.14^bc^
	15	27.56 ± 0.89	10.49 ± 1.96^a^	2.79 ± 1.23^ab^	3.63 ± 0.09^a^
1:4	Uncooked	23.46 ± 0.56	–	–	–
	5	22.43 ± 0.36	2.08 ± 1.67^a^	1.37 ± 0.09^a^	5.48 ± 0.08^a^
	10	22.34 ± 0.62	0.30 ± 0.20^b^	1.59 ± 0.09^b^	5.51 ± 0.11^b^
	15	22.86 ± 0.83	0.73 ± 0.19^a^	1.88 ± 0.21^ab^	5.61 ± 0.14^c^
1:6	Uncooked	20.11 ± 1.24	–	–	–
	5	20.53 ± 1.08	−1.12 ± 1.12^a^	1.31 ± 0.15^a^	6.29 ± 0.03^a^
	10	20.20 ± 0.19	−2.78 ± 1.64^b^	1.03 ± 0.36^b^	6.42 ± 0.27^b^
	15	19.04 ± 0.51	−4.32 ± 1.82^a^	1.55 ± 0.16^c^	6.16 ± 0.12^c^
1:8	Uncooked	17.10 ± 0.60	–	–	–
	5	17.84 ± 0.52	2.34 ± 2.32^a^	1.50 ± 0.12^a^	8.47 ± 0.66^a^
	10	16.43 ± 0.25	−2.75 ± 2.89^b^	1.54 ± 0.09^b^	8.42 ± 0.31^b^
	15	18.18 ± 1.22	−1.29 ± 2.06^c^	1.70 ± 0.03^c^	7.76 ± 1.33^c^
1:10	Uncooked	13.28 ± 0.41	–	–	–
	5	14.28 ± 1.10	−1.23 ± 1.57^a^	1.10 ± 0.07^a^	9.76 ± 0.12^ab^
	10	15.88 ± 0.22	−4.68 ± 2.39^b^	1.19 ± 0.12^b^	8.91 ± 0.13^b^
	15	15.45 ± 0.94	−8.10 ± 2.06^c^	0.85 ± 0.35^ab^	8.84 ± 0.42^a^

*Note*: Data are mean values ± standard deviation. The same letters in the same columns indicate significant differences (*p* < .05). Significant differences were compared within an amaranth flour ratio with the different cooking times.

#### 3.2.3 Cooking characteristics of fresh amaranth pasta

The cooking quality provides information about the characteristics of cooked pasta. Besides texture and color, water absorption (WA), cooking loss (CL), and swelling index (SI) are important quality parameters (Table 2).

The WA of the cooked pasta increased with increasing amaranth flour content within the same cooking times. However, WA increased continuously within the same sample composition as cooking time progressed. In the case of pasta with low amaranth flour content, even water was partially released. This was observed in the 1:10, 1.0% and, 1:6, 1:8, and 1:10 samples, with 1.5% alginate and is a negative value. A correlation between WA and the firmness of these samples can be assumed (*r* = .87). As Kim et al. (2019) found in their study on sodium alginate beads, heat treatment affects the size of the beads. They attribute this to a loss of water from the gel due to syneresis. In this process, the water molecules are pressed out of the gel matrix due to the heat effect of the cooking process. Further, they found that the volume of the beads was reduced by the syneresis effect, and the texture strength increased. In the present study, water loss occurred mainly in the samples that had low amaranth flour contents. The structure of the pasta in these samples was stabilized mainly due to the alginate. Exposure to heat during cooking resulted in syneresis and consequent water loss. Thus, the results agree with those of Kim et al. (2019).

There was a significant difference between the pasta with different amaranth flour contents and the cooking time (*p* < .001). The intensity of WA is related to the structural integrity of the network. A well‐cross‐linked network structure limits the water uptake. This, in turn, is related to the degree of starch degradation and the associated formation of connecting zones by calcium lactate cross‐linked sodium alginate (Lubowa et al., 2020). As in the texture analysis, it can be assumed that the starch in the samples with a high amaranth flour content (1:2) was not completely gelatinized during dough preparation. This is confirmed by the WA with longer cooking time for the samples with a high amaranth flour content. Related to the statement of Lubowa et al. (2020), this means that the structural integrity of the network was not as high at higher amaranth flour contents. The WA was lower when 1.5% alginate was added, as less water was absorbed overall during cooking. There was a significant difference in WA between the 1.0 and 1.5% alginate sample for pasta with 1:2, 1:6, 1:8, and 1:10 (*p* < .001). Also between the alginate contents and the cooking times, a significance was observed (*p* < .001). Lee et al. (2008) attributed the lower water loss in gels with alginate to its polyanionic properties, which limit water mobility. In the present study, this phenomenon occurs in pasta with higher alginate content.

A CL is not desirable in the pasta production and is mainly due to the release or dissolution of solids, such as starch, from the pasta (Lee et al., 2008). The higher the CL, the more unstable the structure becomes. Moreover, a high CL indicates that many minerals and, as mentioned starch, have been washed out, which would reduce the nutritional value and the texture of the pasta (Schoenlechner et al., 2010). Therefore, the CL should be as low as possible (Table 2). When comparing the noodles with different amaranth flour contents in terms of CL, there was a significant difference, especially for the mixture with 1:2 for 1:6 (*p* = .001), 1:8 (*p* = .023), and 1:10 (*p* = .001). In the significance test of CL between the different cooking times, it was found that there was a significant difference between the uncooked and the 5 min cooked noodles (*p* = .010). Each additional cooking time did not result in a significant effect on CL. If the two examined alginate concentrations are compared significantly, no significant correlation (*p* = .314) between the CL data can be detected. Thus, an increased alginate concentration of 1.5% does not result in a decrease in the CL. According to Lubowa et al. (2020), CL is mainly due to the solubility of the loosely bound gelatinized starch on the pasta surface. This, in turn, may depend on the degree of starch gelatinization and the strength of the starch network surrounding the gelatinized starch. In the present study, in addition to the network formation by the gelatinized starch, that of the alginate is also observed. A 15 min cooking time seemed to influence the pasta samples where flour is present in larger amounts: the CL was higher for pasta with 1:2 after 15 min of cooking. The cooking water also exhibited more turbidity after the treatment of these samples than it did for the others. One explanation is that structural stability due to the interaction of starch and alginate gel is not strong enough for 1:2 samples. Here, the loosely bound gelatinized starch leached into the cooking water. Also, an iodine test showed for quick analysis that it was starch in the cooking water (data not shown). The structural integrity of the alginate‐starch network was not strong enough in this sample, as there were too many interference areas due to the high amaranth flour content. However, for no sample composition, a complete disintegration of amaranth pasta was observed, as described in Schoenlechner et al. (2010) for their pasta samples with a high amount of amaranth. All pasta samples had, according to (Hummel, 1966), very good cooking quality (<6% of solids). Compared to amaranth pasta in the study by Schoenlechner et al. (2010), the CL in this study was significantly lower. Lubowa et al. (2020) also found low CL for their rice flour pasta prepared with alginate. Their CL is consistent with the values in this study. The comparatively low CL is due to the hypothesis that the hydrocolloid sodium alginate forms a network around the starch granules, holding them in place during cooking (Hong et al., 2021; Peressini et al., 2018). It can be assumed, that sodium alginate encapsulated and integrated the polysaccharide network, strengthening the structural integrity of the pasta. They explained this by the interaction between sodium alginate and starch, which prevents the dissolution of starch on the surface of the pasta during cooking, resulting in a reduction in the cooking loss of the pasta.

The SI of the pasta is an indicator of the water absorbed by starch gelatinization, hydration of proteins, and water absorption of alginate. The SI of the pasta during cooking is thus an important quality factor and shows whether a volume change has occurred. The SI values in this study showed that both, the amaranth flour, and alginate content influenced the volume increase during cooking (Table 2a,b). Within the same amaranth flour content and increasing cooking time, a significant difference in SI was observed (*p* = .024), thus with longer cooking time, the volume of the pasta increased. There is a correlation between the firmness and the SI of pasta (*r* = .81 for 1.0% and *r* = .72 for 1.5%). This means that during cooking mainly water is absorbed into the pasta, thus softening the texture. This is confirmed by the fact that the SI assumed higher values overall for 1.0% than for the 1.5% samples. However, a simultaneous release of solids is not associated with this, since there is no correlation between the SI and CL.

## 4 CONCLUSION

Amaranth has special nutritional and health‐promoting properties that could make it a ingredient in the manufacture of gluten‐free products. The results of our study showed that pregelatinized whole‐grain amaranth flour with alginate in combination with calcium ions can be successfully formed into a water‐ and thermostable gel. The fresh amaranth pasta prepared in this way has good dough processing properties as well as cooking properties. It was found that a higher alginate content of 1.5% positively affected the doughs and pasta. In particular, the blends with an amaranth flour/water ratio of 1:4 and 1:6 with 1.5% alginate by weight proved to be the best combination. In these samples, the amaranth flour content was high, and the pasta properties, such as firmness and color, were appealing. Also, during cooking, there was no loss of quality due to water absorption, cooking loss, and swelling index appearing in these samples. This was especially for 1:6, 1.5% sample. This study shows that alginate has positive properties to produce gluten‐free pasta from amaranth and can hold a binary system of gelatinized starch and denatured proteins together.

## FUNDING INFORMATION

The results were obtained as part of a research project (grant number KF2254613SK4) funded by the German Federal Ministry for Economic Affairs and Energy via Industrielle Gemeinschaftsforschung (AiF).

## CONFLICT OF INTEREST STATEMENT

The authors have no conflict of interest to declare.

## Data Availability

Data are available on request due to privacy/ethical restrictions.
